# Characterization and genome sequence of N,N-dimethylformamide degradation in *Paracoccus sulfuroxidans* DM175A1-1 isolated from activated sludge of wastewater

**DOI:** 10.3389/fmicb.2024.1419461

**Published:** 2024-08-26

**Authors:** Gang Zheng, Yue Su, Wenwu Zhang, Xingping Liu, Li Shao, Zhibo Shen, Dongdong Zhang, Kaiyang Wang, Zhoudi Miao

**Affiliations:** ^1^Institute of Marine Biology and Pharmacology, Ocean College, Zhejiang University, Zhoushan, China; ^2^Xianghu Laboratory, Hangzhou, China; ^3^Trend Biotech Co., Ltd., Hangzhou, China; ^4^Zhoushan Sewerage Treatment Co., Ltd., Zhoushan, China; ^5^Environmental Sanitation Management Office, Zhoushan, China; ^6^Ocean Research Center of Zhoushan, Zhejiang University, Zhoushan, China

**Keywords:** N,N-dimethylformamide, *Paracoccus sulfuroxidans*, wastewater treatment, efficient degradation, biological method

## Abstract

N,N-dimethylformamide (DMF) is an organic solvent with stable chemical properties and high boiling point. Based on its good solubility, DMF is widely used in synthetic textile, leather, electronics, pharmaceutical and pesticide industries. However, the DMF pollutes the environment and does harm to human liver function, kidney function, and nerve function. Herein, an efficient DMF-degrading strain, DM175A1-1, was isolated and identified as *Paracoccus sulfuroxidans*. This strain can use DMF as the sole source of carbon and nitrogen. Whole-genome sequencing of strain DM175A1-1 revealed that it has a 3.99-Mbp chromosome a 120-kbp plasmid1 and a 40-kbp plasmid2. The chromosome specifically harbors the *dmfA1* and *dmfA2* essential for the initial steps of DMF degradation. And it also carries the some part of genes facilitating subsequent methylotrophic metabolism and glutathione-dependent pathway. Through further DMF tolerance degradation experiments, DM175A1-1 can tolerate DMF concentrations up to 10,000 mg/L, whereas the majority of *Paracoccus* strains could only show degradation activity below 1,000 mg/L. And the efficiency of organic nitrogen conversion to NH_3_-N in DMF can reach 99.0% when the hydraulic retention time (HRT) is controlled at 5 days. Meanwhile, it showed a significant degradation effect at a pharmaceutical enterprise in Zhejiang Province with high concentration of DMF wastewater. This study provides a new strain *Paracoccus sulfuroxidans* DM175A1-1 which shows a significant influence on DMF degradation, and reveals the characterization on its DMF degradation. It lays the foundation for the application of biological method in the efficient degradation of DMF in industrial wastewater.

## Introduction

N,N-dimethylformamide (DMF) is a common multifunctional solvent that is widely used in pesticide, pharmaceutical, petrochemical, and other production industries (Ding and Jiao, 2012; Bromley-Challenor et al., 2000; Nisha et al., 2015). In recent years, with the expansion of the scale of chemical production, DMF has aggravated the pollution of the environment with the increase of industrial wastewater (Chen et al., 2016). DMF can enter the human body through breathing and skin contact, which has greater harm to human liver function, kidney function, and nerve function (Wang et al., 2014; Li and Zeng, 2019). At present, there are physical, chemical, and biological methods for treating DMF wastewater, and biological processes have been applied in industrial wastewater treatment because of their advantages of low cost and low pollution (Das et al., 2006; Sun et al., 2008; Dziewit et al., 2010). When the concentration of pollutants in wastewater is high, it will influence the growth and degradation effect of microorganisms (Astals et al., 2015). At present, the treatment of the DMF wastewater is mainly relying on activated sludge. However, the activated sludge could only tolerant about 200 mg/L DMF concentration compared to the 2,000 mg/L DMF concentration of factory wastewater (Zhou et al., 2018; Astals et al., 2015). The low degradation rate of DMF will cause the exceedance of total nitrogen released, which increase the difficulty in subsequent treatment of nitrogen content. Hence, it needs to be domesticated and isolated from the environment to get some strains that can degrade DMF efficiently, which is essential for practical industrial production applications.

Several bacteria that degrade DMF have been isolated and identified, such as *Alcaligenes* (Hasegawa et al., 1997), *Paracoccus* (Nisha et al., 2015; Doronina et al., 1998; Czarnecki et al., 2017), *Pseudomonas* (Ghisalba et al., 1985), *Methylobacterium* (Doronina et al., 2000), *Mycobacterium* (Urakami et al., 1990), *Ochrobactrum* (Veeranagouda et al., 2006), etc. *Paracoccus* is the main genus for degrading DMF, and there are several *Paracoccus* strains were reported that have the capability of degrading DMF effectively (Zhou et al., 2018; Astals et al., 2015). Swaroop et al. (2009) found that the *Paracoccus* sp. strain DMF bacteria would briefly accumulate dimethylamine and methylamine during the growth process using DMF, which would eventually be converted into ammonia and carbon dioxide and degrade DMF.

In previous study, they found that some of the strains *Paracoccus aminophilus* JCM 7686 and *Methylobacterium* sp. Strain DM1 contained enzymes that could degrade DMF, named DMFase (Dziewit et al., 2010; Lu et al., 2019). In contrast, others did not include this enzyme, so they speculated that there might be two pathways of DMF degradation. The critical enzyme in one of the pathways was DMFase, which degraded DMF to dimethylamine (DMA) and formic acid (Dziewit et al., 2010). Subsequently, DMA forms methylamine and formaldehyde by the action of dimethyl dehydrogenase. Then methylamine will form ammonia and formaldehyde by the activity of methylamine dehydrogenase (Ghisalba et al., 1985; Veeranagouda et al., 2006). Another pathway of DMF degradation is achieved through repeated oxidative demethylation of DMF, which is speculated to be possessed by some *Pseudomonas* species (Hans-Peter et al., 2010). The two methyl groups on N are removed by methyl formamide oxidase and formamide oxidase, respectively, to form formamide, which is further hydrolyzed by formamidase to produce ammonia and formic acid (Ghisalba et al., 1985). The DMF degradation mechanisms of different genera may differ significantly, and there are relatively few studies on DMF degradation mechanisms in terms of genes. Therefore, it is essential to identify the DMF degradation mechanisms of *Paracoccus sulfuroxidans*.

In this study, we isolated four efficient DMF degrading strains by acclimatization, which were able to utilize DMF as the sole carbon and nitrogen source. Among them, DM175A1-1 showed the best degradation effect, and the whole gene sequencing data of this strain were analyzed to elucidate its relevant genes and degradation pathways responsible for DMF degradation. The DM175A1-1 can achieve efficient ammonification of DMF, which could solve the difficulty of converting organic nitrogen to ammonia nitrogen during the degradation then, the nitrogen removal can be achieved in combination with nitrification or denitrification processes subsequently. Finally, in order to enhance the tolerance of the high DMF concentration about DM175A1-1, we further investigated its degradation ability by tolerance test.

## Materials and methods

### Culture medium

The inorganic salt medium with DMF as the sole source of carbon and nitrogen (1.2 g/L KH_2_PO_4_, 6.8 g/L K_2_HPO_4_, 0.5 g/L NaCl, 0.1 g/L MgSO_4_·7H_2_O, 0.1 g/L MnSO_4_·H_2_O, 0.1 g/L CaCl_2_, 0.1 g/L FeSO_4_·7H_2_O, 0.006 g/L Na_2_MoO_4_·2H_2_O, 0.006 g/L CuSO_4_·5H_2_O, 0.007 g/L ZnSO4·7H_2_O, 0.0001 g/L CoCl_2_·6H_2_O, 0.0124 g/L H_3_BO_3_, 0.00001 g/L Vitamin B1, pH 6.0) was used to domesticate the isolates. LB medium as the seed solution medium for strains. DMF1 medium as the purification medium for strains (additional 0.2 g/L DMF and 0.2 g/L dimethylamine were added on top of the ingredients in LB medium).

### Isolation and identification of domesticated strains

Fresh sludge of 10 mL from each pharmaceutical plant was inoculated into a triangular flask containing 100 mL of inorganic salt medium (DMF concentration of 1,000 mg/L) and incubated in an incubator at 30°C with 130 r/min oscillation, during which the total nitrogen (TN) and NH_3_-N values in the system were sampled every one day. The culture was incubated until the ammonification rate of TN reached more than 90% (ammonification refers to the conversion of organic nitrogen in TN into NH_3_-N), then transferred to fresh inorganic salt medium (DMF concentration of 2,000 mg/L) at 10% inoculum, monitored the TN and NH_3_-N values in the system and continued to incubate. The DMF concentration was gradually increased to 5,000 mg/L by transferring five times consecutively.

One millilitre of bacterial broth from the culture system with good growth condition of the strain was taken for high-throughput sequencing. At the same time, the above bacteria were at a dilution of 10^−2^ to 10^−8^ spread onto a solid medium containing 1,000 mg/L DMF and incubated at 30°C for 5 days single colonies were picked into DMF1 medium, incubated at 30°C for 3 days, and then purified by scribing, and the single purified strains were identified by DNA sequencing. The morphology of the bacterium was observed by light microscopy and cultured with Petri dishes. The isolated and purified strains were subjected to Gram staining and physiological and biochemical characterization experiments, and the identification results of 16S rDNA were used as the species name.

### Genome sequencing and genetic information analysis

The version of MEGA was MEGA X. The evolutionary using the Neighbor-Joining method, Kimura 2-parameter method, Numbers at branch points indicate bootstrap percentages (based on 1,000 replications). Only values above 50% are shown. Bar, 0.01 substitutions per nucleotide position. The 16S rRNA gene sequence of *Tropicibacter naphthalenivorans* CECT 7648 was used as the outgroup.

Genomic DNA was extracted using the Wizard genomic DNA purification kit (product no. A1125; Promega). Whole-genome sequencing was performed on the Illumina MiSeq and PacBio sequencing platforms. Functional gene predictions and annotations were performed using GeneMarkS,^1^ the Rapid Annotation Subsystem Technology (RAST) database, BLAST,^2^ and UniProt.^3^ Insertion elements were predicted using Isfinder.

### Degradation of the DMF

The strains were amplified and cultured in LB medium until the OD600 reached about 1.5, centrifuged at 6,000 r/min for 3 min, the supernatant was discarded, and the pellet was used further. The pellet was then resuspend with sterile distilled water to prepare the seed solution. The feed water was an inorganic salt medium containing 5,000 mg/L DMF, adjusted pH = 6.0–7.0, which was artificially configured wastewater. The inorganic salt medium was added to the strain treatment tank, adjusted pH = 6.0–7.0, and various sub-liquids were inoculated in the strain treatment tank according to 20% inoculum, and granular activated carbon was added as the strain carrier to enrich and retain the strain. The hydraulic retention time (HRT) of the influent water in the strain treatment tank was controlled to be 3 days, dissolved oxygen (DO) >2.0 mg/L, and water temperature 30–35°C. The primary water quality data in the system were monitored daily, such as COD, NH_3_-N, and TN.

### Statistical analysis

Every group of the experiments repeated for 4 times, *n* = 4. Data were expressed as the mean ± standard error of the mean (SEM), visualized using GraphPad Prism (8.3.0), one-way analysis of variance (ANOVA) of SPSS (version 25.0), and Dunnett’s multiple comparison test for statistical analysis, with significant differences between groups (*p* < 0.05).

## Results and discussion

### The acclimatization and isolation of the strains

Fresh sludge was used for acclimatization, in which the strains were cultured in an inorganic medium with a DMF concentration of 1,000 mg/L for 1 week, the NH_3_-N in the system increased significantly to about 160 mg/L, and the TN ammonification rate reached more than 90%. Four strains of bacteria with DMF degradation effect were obtained by acclimatization culture.

**Figure 1:**
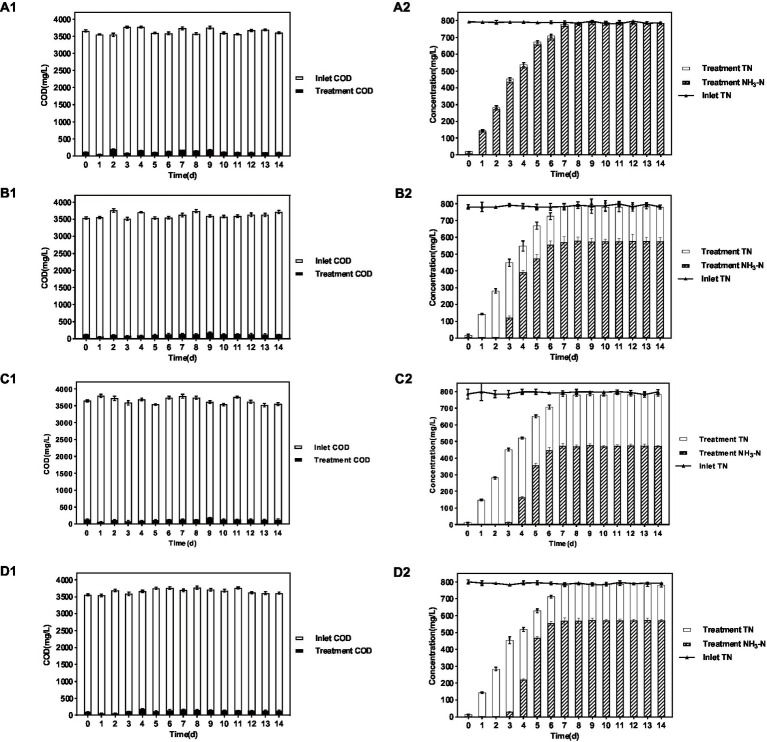
Treatment effect of 4 strains on various indicators of wastewater. **(A–D)** Represent *P. sulfuroxidans* DM175A1-1, *A. lusatiense* 3-1-1, *G. flavus* 2-CZ-5, *S. paucimobilis* 3-3-10, respectively. The number 1 after alphabet represents the COD treatment effect of the strain, number 2 represents the TN treatment effect of the strain and the ammonification effect. Values were displayed as the mean ± SEM, *n* = 4; the difference between groups was statistically significant (*p* < 0.05).

As shown in the results of Figure 1 and Table 1, in the group system of *P. sulfuroxidans* DM175A1-1, both NH_3_-N and TN showed a slowly increasing trend in the first 6 days, and the proportion of NH_3_-N to TN was basically above 95%; as shown in Table 1, after the 7th day, both NH_3_-N and TN data in the treated system were stable at about 780 mg/L, and the proportion of NH_3_ to TN was at above 98%. In the system of the *A. lusatiense* 3-1-1, after the 7th day, the NH_3_-N data in the treatment system were stable at about 575 mg/L, the TN data were about 780 mg/L, and the proportion of NH_3_-N to TN was maintained at about 74%.

**Table 1:** Treatment effect of 4 strains on various indicators of wastewater.

Index	*P. sulfuroxidans* DM175A1-1	*A. lusatiense* 3-1-1	*G. flavus* 2-CZ-5	*S. paucimobilis* 3-3-10
Inlet COD (mg/L)	3,639	3,616	3,655	3,665
Treatment COD (mg/L)	130	139	139	146
Removal rate of COD	96.4%	96.2%	96.2%	96.0%
Inlet TN (mg/L)	790	786	793	791
Treatment TN (mg/L)	786	781	782	786
Treatment NH_3_-N (mg/L)	786	577	473	571
Ammonification rate	100%	73.8%	60.4%	72.7%

Values were displayed as the mean ± SEM, *n* = 4; the difference between groups was statistically significant (*p* < 0.05).

In the *G. flavus* 2-CZ-5 experimental system, after the 7th day, the NH_3_-N data in the treated system were stable at about 473 mg/L, the TN data at about 780 mg/L, and the proportion of NH_3_-N to TN was maintained at about 60%. in the *S. paucimobilis* 3-3-10 experimental system, after the 7th day, the NH_3_-N data in the treated system were stable at about 575 mg/L, the TN data at about 780 mg/L, and the proportion of NH_3_-N to TN was maintained at about 60%. The NH_3_-N data were stable at about 572 mg/L, and the TN data were about 780 mg/L, and the proportion of NH_3_-N to TN was maintained at about 73%.

The experimental results of DMF degradation by single bacteria showed that in the four single bacteria experimental systems, all strains could take advantage of DMF effectively and their OD600 is over 1.50, pH in all four systems increased significantly to more than 8.0, and a certain amount of acid should be added daily to adjust pH to about 6. The DMF degradation effect of *P. sulfuroxidans* DM175A1-1 was better than the other three monocultures, and the DMF degradation rate reached more than 90%.

### Identification of the strains

**Figure 2:**
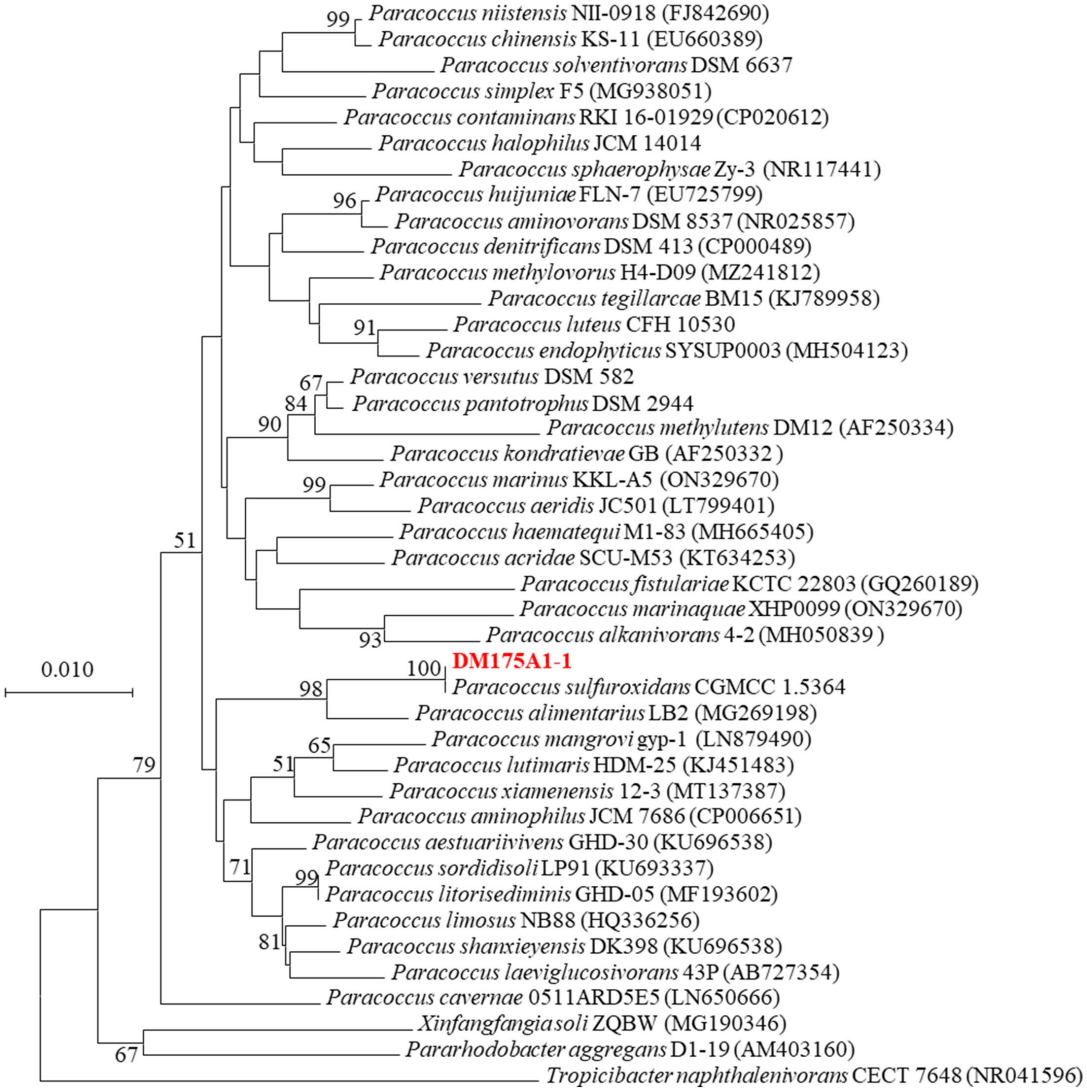
Phylogenetic tree of strain *P. sulfuroxidans* DM175A1-1.

After 3 days incubation on LB Petri dishes, the colony morphology was round and raised. The colony surface was smooth and moist. The edge was neat and beige in color, Gram negative, and the physiological and biochemical characteristics were initially identified as *Paracoccus*. 16S rDNA sequence comparison, the similarity between strain DM175A1-1 and *Paracoccus sulfuroxidans* CGMCC 1.5364T was 99%, so the phylogenetic tree was constructed by MEGA software, and strain DM175A1-1 was also closely clustered with *Paracoccus sulfuroxidans* CGMCC 1.5364T. As shown in Figure 2, the phylogenetic tree was constructed by MEGA software, and strain DM175A1-1 was also closely clustered with *Paracoccus sulfuroxidans* CGMCC 1.5364T, so it was identified as *Paracoccus sulfuroxidans* strain.

### Multireplicon genome of strain DM175A1-1

**Figure 3:**
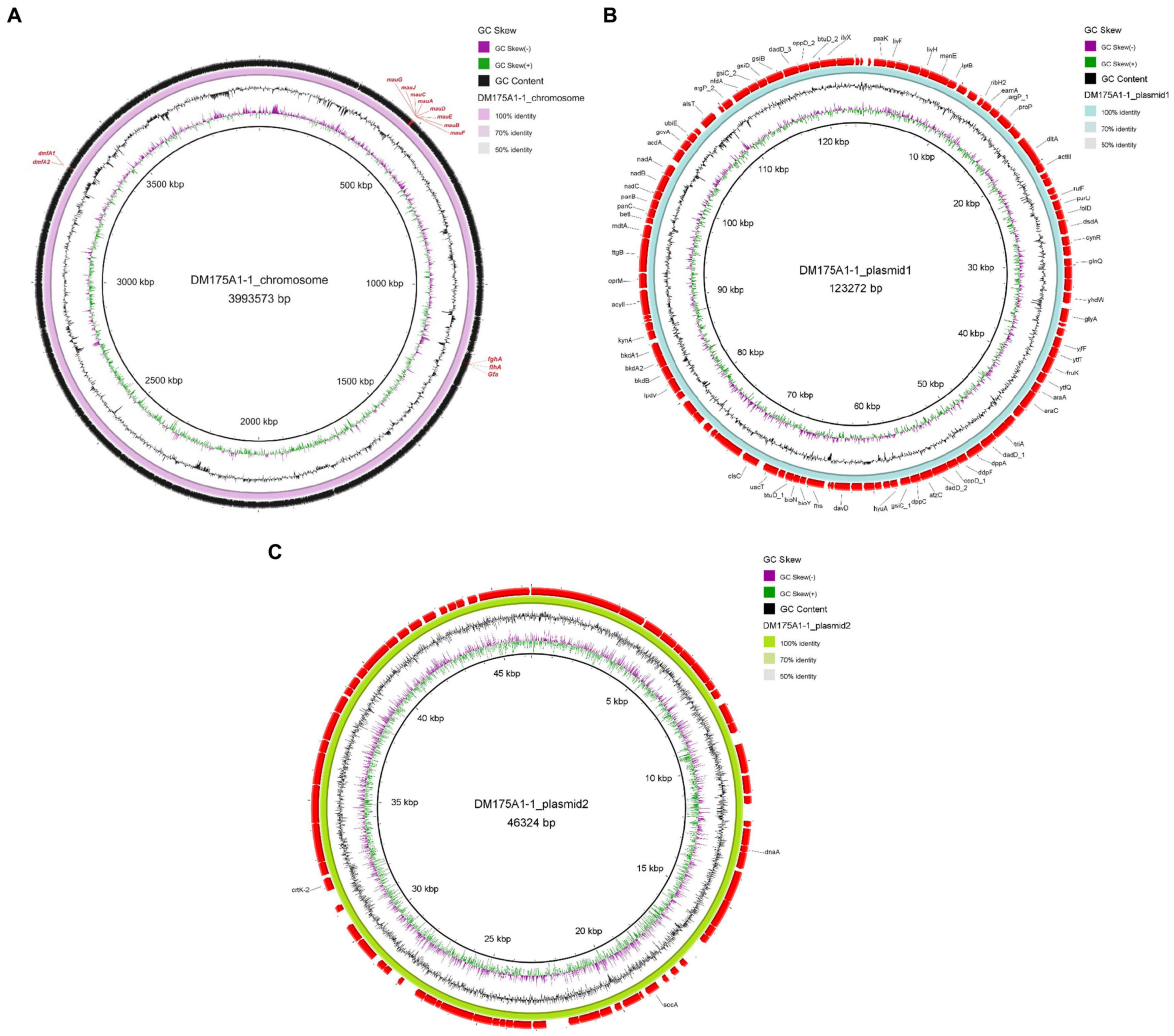
Genomic analysis revealing the circular replicons in the genome of strain DM175A1-1. **(A)** The chromosome. **(B)** The plasmid1. **(C)** The plasmid2.

Whole-genome sequencing was performed, and analysis of the data revealed that the genome of strain DM175A1-1 has a multireplicon structure, with a single circular chromosome and two plasmids (Figure 3). The circular chromosome is 3,993,573 bp in size; the plasmid1 is 123,272 bp in size; and the plasmid2 is 46,324 bp in size. The whole length of the genome sequence is 4,163,169, with a G + C content of 64.40%. After the analysis of the function, the CDS results showed that the Gene number is 4,139, and the gene total length is 3,800,551 bp, with a coding percentage of 91.3% (Table 2).

**Table 2:** Gene information statistics.

Sample	DM175A1-1
Gene number	4,139
Gene total length (bp)	3,800,551
Gene average length (bp)	918
Gene density (gene number/Kb)	0.994
GC content in gene region (%)	64.9
Gene/geonme (%)	91.3
Intergenetic region length (bp)	362,618
GC content in intergenetic region (%)	58.8
Intergenetic length/genome (%)	8.71

We submitted the genome of the DM175A1-1 to the blast server. *P. aminovorans* JCM 7685, which has a complete DMF degradation pathway, carries two genes, *dmfA1* and *dmfA2*, responsible for encoding the DMFase that initially degrades DMF to DMA and MA (Czarnecki and Bartosik, 2019). The DM175A1-1 also has the *dmfA1* and *dmfA2* genes encoding the DMFase, as shown in Figure 3, so the initial pathway of DM175A1-1 to degrade DMF to DMA and MA may be the same as that of the *P. aminovorans* JCM 7685. Then *P. aminovorans* JCM 7685 degrades DMA to formate by the NMG pathway, in which key genes in the NMG pathway are not found in DM175A1-1, so it was hypothesised that other DMA degradation pathways may exist in DM175A1-1 (Czarnecki and Bartosik, 2019). The MA degradation pathway has been reported in *Paracoccus* spp. *P. denitrificans* Pd 1222, MA is degraded to formaldehyde by the action of MA dehydrogenase encoded by *mauA* and *mauB*, and *P. denitrificans* Pd 1222 oxidises formaldehyde to formate by a glutathione-dependent pathway requiring three enzymes, namely S-(hydroxymethyl) glutathione synthetase (*gfa*), S-(hydroxymethyl) glutathione synthase, S (hydroxymethyl) glutathione dehydrogenase (*flhA*) and S-formylglutathione hydrolase (*fghA*) (Czarnecki and Bartosik, 2019; Lu et al., 2019). As shown in Figure 3, the chromosome of DM175A1-1 carries the *mau* gene cluster encoding formaldehyde dehydrogenase, where the cluster contains *mauA* and *mauB*, and three genes in the pathway for further breakdown of formaldehyde to formate, as shown in the Figure 3, *flhA*, *fghA* and *gfa*, respectively. Therefore, the product of the initial degradation of DM175A1-1, MA, can be converted to formaldehyde through the MA dehydrogenase encoded by *mauA* and *mauB*, which in turn is oxidised to formate through the glutathione-dependent pathway. As DM175A1-1 contains some of the genes in the DMF degradation pathway that have been studied, but also lacks some of the genes in the degradation pathway, new degradation pathways may exist in DM175A1-1 and are the next direction for further research.

### Tolerance test of the *Paracoccus sulfuroxidans* DM175A1-1

To further verify the practical effect of strain DM175A1-1, the DMF concentration in the influent water was increased to 10,000 mg/L for the experiment. The experimental procedure was the same as 1.4.2. The system without the strain was set as the blank group, and the system with the activated sludge (MLVSS of 6,000 mg/L) was set as the control group, and the treatment effects of HRT 3 days and 5 days were investigated. The purpose was to check the tolerance of the strain *P. sulfuroxidans* DM175A1-1 to DMF and compare it with the treatment effect of activated sludge.

**Figure 4:**
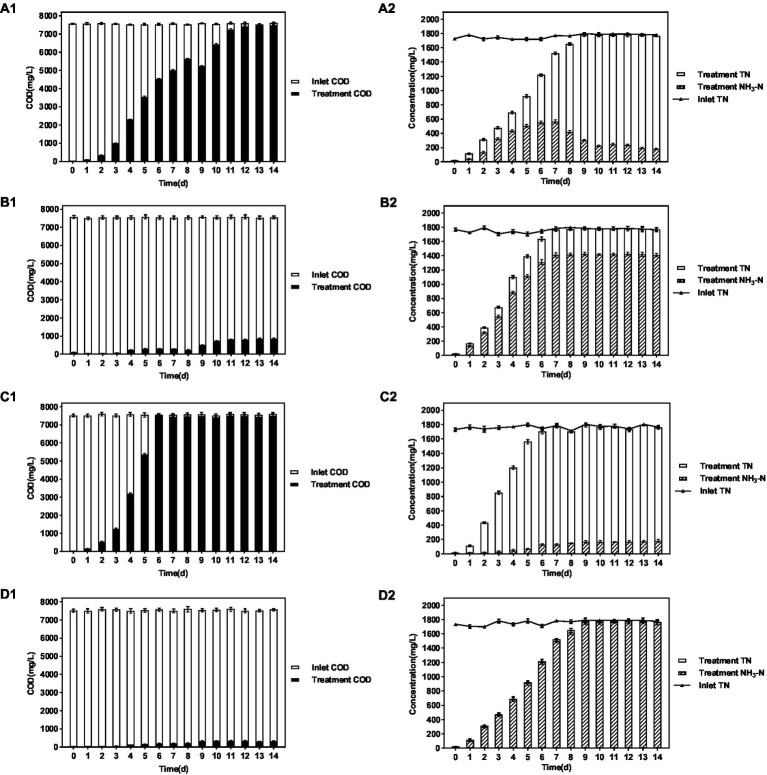
Effect of *P. sulfuroxidans* DM175A1-1 on the treatment of high concentration DMF. **(A–D)** Space Team (HRT 3 days), Action Pollution Team (HRT 3 days), *P. sulfuroxidans* DM175A1-1 Team (HRT 3 days), *P. sulfuroxidans* DM175A1-1 Teams (HRT 5 days), 1.2 represents the COD treatment effect of the strain, the TN treatment effect of the strain, and the ammonification effect. Values were displayed as the mean ± SEM, *n* = 4; the difference between groups was statistically significant (*p* < 0.05).

The results are shown in Figure 4 and Table 3. When the influent DMF concentration reached 10,000 mg/L, the activated sludge group decreased in the late ammonification period, probably due to poisoning, and the final ammonification rate was as low as 14.6%. In contrast, the *P. sulfuroxidans* DM175A1-1 strain treatment group (groups C and D) had apparent growth and better activity in the treatment system, with specific higher COD removal capacity and ammonification capacity, and when the HRT was 3 days, the COD removal rate was 91.0%, and ammonification rate was 80.0%; when the HRT was extended to 5 days, the COD removal rate continued to rise to 91.0% and When the HRT was extended to 5 days, the COD removal rate continued to increase to 91.0%, and the ammonification rate reached 99.0%. In conclusion, *P. sulfuroxidans* DM175A1-1 can tolerate DMF concentrations up to 10,000 mg/L, and the conversion efficiency of organic nitrogen to NH_3_-N in DMF can reach 99.0% when the HRT is controlled at 5 days.

**Table 3:** Effect of *P. sulfuroxidans* DM175A1-1 on the treatment of high concentration DMF.

Index	A	B	C	D
Inlet COD (mg/L)	7,555	7,550	7,547	7,542
Treatment COD (mg/L)	7,468	6,695	680	308
Removal rate of COD	1.2%	11.3%	91.0%	95.9%
Inlet TN (mg/L)	1,768	1,786	1,781	1,785
Treatment TN (mg/L)	1,758	1,758	1,775	1,758
Treatment NH_3_-N (mg/L)	182	257	1,420	1,740
Ammonification rate	9.6%	14.6%	80.0%	99.0%

Values were displayed as the mean ± SEM, *n* = 4; the difference between groups was statistically significant (*p* < 0.05).

### The treatment of the wastewater based on *Paracoccus sulfuroxidans* DM175A1-1

The wastewater came from a pharmaceutical enterprise in Zhejiang, with a DMF content of about 1.2% (w/v), a water volume of 60 t/d, and wastewater quality, as shown in Table 4. In order to reduce the introduction of nitrogenous substances from the strain medium into the wastewater, the centrifuges are not provided in the factory, so the strain was fermented with 1/5 concentration of LB medium and was pitched when the fermentation reached about 1.5 OD600 value. The wastewater was supplemented with KH_2_PO_4_ 0.5 g/L, and H_2_SO_4_ was used to adjust the pH of the wastewater = 6.0.

**Table 4:** Basic water quality of high concentration DMF wastewater.

COD (mg/L)	NH_3_-N (mg/L)	TP (mg/L)	NO^2−^-N (mg/L)	NO^3−^-N (mg/L)	TN (mg/L)	pH	TDS
10,000	125	0	0	0	2,000	10	0.03%

The device is used to handle the wastewater, a filler layer is set at 1/3 of the distance from the bottom of the device, and the filler is preferably granular activated carbon, with a dosage of 10–13% (the volume ratio of filler to the device). The aerobic process was adopted, controlling HRT about 5 days, DO> 2 mg/L, water temperature 30–33°C, and 20% of strain injection. During the strain treatment, the pH of the strain treatment pool will rise to 9.0, and H_2_SO_4_ needs to be added to consistently control the pH of the strain treatment pool = 6.0–7.0 (optimal 6.0). When the treatment effect is not good, additional carbon sources such as glucose 2–3 g/L can be supplemented or supplemented strains. The primary water quality data in the system were monitored daily.

**Figure 5:**
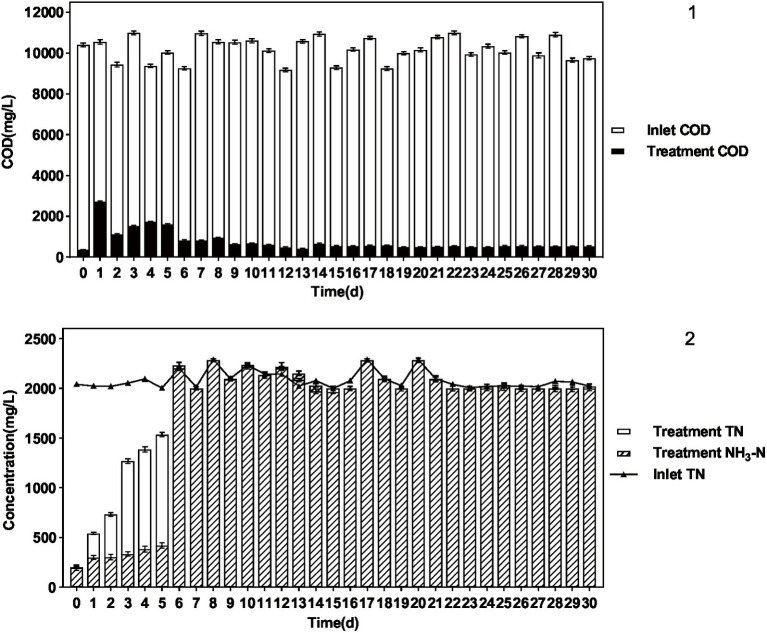
Treatment effect of *P. sulfuroxidans* DM175A1-1 on high concentration DMF wastewater. (1) The COD treatment effect of the strain. (2) The TN treatment effect of the strain and the ammonification effect. Values were displayed as the mean ± SEM, *n* = 4; the difference between groups was statistically significant (*p* < 0.05).

The results are shown in Figure 5 and Table 5. *P. sulfuroxidans* DM175A1-1 strain treated wastewater with DMF content up to 1.2% and TN about 2,000 mg/L well, and the COD removal rate was nearly 95%, which could basically achieve mineralization; DMF removal rate reached 100%, and combined with COD data, it was presumed that the remaining material was the intermediate product in the process of DMF degradation; TN Basically, all of the TN was converted into NH_3_-N, and the ammonification rate reached 99.89%.

**Table 5:** Treatment effect of *P. sulfuroxidans* DM175A1-1 on high concentration DMF wastewater.

No.	Index	Data
1	Inlet COD (mg/L)	10,232
	Treatment COD (mg/L)	531
	Removal rate of COD	94.81%
2	Inlet DMF (mg/L)	11,700
	Treatment COD (mg/L)	0
	Removal rate of COD	100%
3	Inlet TN (mg/L)	2,079
	Treatment TN (mg/L)	2,058
	Treatment NH_3_-N (mg/L)	2,056
	Ammonification rate	99.89%

Values were displayed as the mean ± SEM, *n* = 4; the difference between groups was statistically significant (*p* < 0.05).

## Conclusion

In this study, the DM175A1-1 was identified as *Paracoccus sulfuroxidans*, which were growing with DMF as the only carbon and nitrogen source. Whole-genome sequencing of strain DM175A1-1 revealed that it has a 3.99-Mbp chromosome a 120-kbp plasmid1 and a 40-kbp plasmid2. Through the comparison of the genome to others, we found that the chromosome has the key genes in the initial phase of methylotrophy pathways in the genus *Paracoccus*. Then after the further DMF tolerance degradation experiments, DM175A1-1 can tolerate DMF concentration up to 10,000 mg/L, and the efficiency of organic nitrogen conversion to NH_3_-N in DMF can reach 99.0% when the HRT is controlled at 5 days. Meanwhile, the application experiments were conducted for a pharmaceutical enterprise in Zhejiang Province with a high concentration of DMF wastewater, and the wastewater treatment effect was good. The prevailing treatment methods for DMF-containing wastewater are typically biochemical, which are frequently costly and environmentally benign. At present, there are few applications of microbial degradation in industry. This study has successfully demonstrated the degradation of DMF in enterprise applications, providing insights and support for future research. As the specific degradation pathway of this strain has not yet been fully elucidated, future research will focus on elucidating the mechanism and mechanism. This will further deepen the connection between the genome and degradation products, and determine the subsequent degradation mechanism and pathway.

## Data Availability

The datasets presented in this study can be found in online repositories. The names of the repository/repositories and accession number(s) can be found in the article/Supplementary material.

## Footnotes

^1^http://exon.gatech.edu/GeneMark

^2^https://www.ncbi.nlm.nih.gov/BLAST

^3^www.uniprot.org
