# Supplementary material for: Characterization and genome sequence of N,N-dimethylformamide degradation in Paracoccus sulfuroxidans DM175A1-1 isolated from activated sludge of wastewater
Source: Front Microbiol. 2024 Aug 26;15:1419461. doi: 10.3389/fmicb.2024.1419461 (PMC11381386; doi:10.3389/fmicb.2024.1419461)
Supplement: Supplementary file 1 [file Data_Sheet_1.PDF]

Supplementary material

Characterization and genome sequence of N,N-Dimethylformamide degradation in Paracoccus sulfuroxidans DM175A1-1 isolated from activated sludge of wastewater

Gang Zheng[1,2], Yue Su[4], Wenwu Zhang[4], Xingping Liu[5], Li Shao[5], Zhibo Shen[6], Dongdong Zhang[1], Kaiyang Wang[3] and Zhoudi Miao[3]*

[1] Institute of Marine Biology and Pharmacology, Ocean College, Zhejiang University, Zhoushan, China,

[2] Xianghu Laboratory, Hangzhou, China,

[3] Ocean Research Center of Zhoushan, Zhejiang University, Zhoushan, China,

[4] Trend Biotech Co., Ltd., Hangzhou, China,

[5] Zhoushan Sewerage Treatment Co., Ltd., Zhoushan, China,

[6] Environmental sanitation management office, Daishan, Zhoushan, China

*Corresponding authors: Zhoudi Miao

E-mail addresses: 489664306@qq.com

Tables S1. Strain information

Name	Collection number
DM175A1-1	CGMCC No. 23659

Tables S2. The indentification results of 16S rDNA

Name	Top-hit taxon	Top-hit strain	Similarity（%）
DM175A1-1	*Paracoccus sulfuroxidans*	CGMCC 1.5364	99.04

Tables S3. The sequence information of 16S rDNA

Name	Sequence (5’-3’)
DM175A1-1	TTGGGCACTCTGGAAGAACTGCCGATGATAAGTCGGAGGAAGGTGTG<br>GATGACGTCAAGTCCTCATGGCCCTTACGGGTTGGGCTACACACGTGC<br>TACAATGGTGGTGACAGTGGGTTAATCCCCAAAAGCCATCTCAGTTCG<br>GATTGGGGTCTGCAACTCGACCCCATGAAGTTGGAATCGCTAGTAATC<br>GCGGAACAGCATGCCGCGGTGAATACGTTCCCGGGCCTTGTACACAC<br>CGCCCGTCACACCATGGGAGTTGGTTCTACCCGACGGCCGTGCGCTA<br>ACCTTTGGAGGCAGCGACCACGTAGATACGC
